# Comparison of adjunctive use of aripiprazole with bupropion or selective serotonin reuptake inhibitors/serotonin–norepinephrine reuptake inhibitors: analysis of patients beginning adjunctive treatment in a 52-week, open-label study

**DOI:** 10.1186/1756-0500-7-459

**Published:** 2014-07-18

**Authors:** Anita H Clayton, Ross A Baker, John J Sheehan, Zachary J Cain, Robert A Forbes, Sabrina Vogel Marler, Ronald Marcus, Robert M Berman, Michael E Thase

**Affiliations:** 1 Department of Psychiatry and Neurobehavioral Sciences, The University of Virginia, Charlottesville, 2955 Ivy Rd, Northridge Suite 210, Charlottesville VA 22903, USA; 2 Otsuka Pharmaceutical Development & Commercialization, 1 University Square Drive, Princeton NJ 08540, USA; 3 Bristol-Myers Squibb, Plainsboro, 777 Scudders Mill Road, Plainsboro NJ 08536, USA; 4 Bristol-Myers Squibb, Wallingford, CT, USA; 5 Department of Psychiatry, University of Pennsylvania School of Medicine, 3535 Market Street, Suite 670, Philadelphia, PA 19104, USA

**Keywords:** Adjunctive antidepressant, Atypical antipsychotic, Aripiprazole, Bupropion, Dopamine, Major depressive disorder, Selective serotonin reuptake inhibitors, Serotonin–norepinephrine reuptake inhibitors, Sexual dysfunction

## Abstract

**Background:**

This post hoc analysis assessed the safety, tolerability and effectiveness of long-term treatment with aripiprazole adjunctive to either bupropion or selective serotonin reuptake inhibitors (SSRIs)/serotonin–norepinephrine reuptake inhibitors (SNRIs) in patients with major depressive disorder (MDD).

**Methods:**

Data from de novo patients (did not participate in 2 previous studies) in a 52-week, open-label safety study of adjunctive aripiprazole after documented inadequate response to 1–4 antidepressant treatments (ADTs; SSRI, SNRI, or bupropion) were analyzed post hoc. Assessments included safety and tolerability, sexual functioning (Massachusetts General Hospital Sexual Functioning Inventory [MGH-SFI]) and Clinical Global Impressions–Severity (CGI-S).

**Results:**

Forty-seven patients received bupropion plus aripiprazole and 245 received an SSRI/SNRI plus aripiprazole; 19 (40.4%) and 78 (31.8%), respectively, completed 52 weeks of treatment, and 46 and 242, respectively, received ≥1 dose of study medication (safety sample). Median time to discontinuation (any reason) was 184.0 days. Overall, 97.8% of patients in the bupropion group and 93.8% in the SSRI/SNRI group experienced ≥1 adverse event. The most common treatment-emergent adverse events were fatigue (26.1%) and somnolence (21.7%) with bupropion and fatigue (23.6%) and akathisia (23.6%) with an SSRI/SNRI. Mean change in body weight at week 52 (observed cases) was +3.1 kg for bupropion and +2.4 kg for an SSRI/SNRI. Treatment-emergent, potentially clinically relevant abnormalities in fasting glucose occurred in 8.3% of patients with bupropion and 17.4% with an SSRI/SNRI; for abnormalities in fasting total cholesterol, the incidence was 25.0% and 34.7%, respectively. Mean (SE) change from baseline in fasting glucose was 1.4 (1.9) mg/dL with bupropion and 2.7 (1.5) mg/dL with an SSRI/SNRI. Baseline MGH-SFI item scores indicated less severe impairment with bupropion versus an SSRI/SNRI; in both groups most MGH-SFI items exhibited improvement at week 52. Mean CGI-S improvement at week 52 (last observation carried forward) was -1.4 with bupropion and -1.5 with an SSRI/SNRI (efficacy sample).

**Conclusions:**

There were no unexpected AEs with long-term adjunctive aripiprazole therapy when added to either bupropion or SSRIs/SNRIs, and symptom improvement was similar between ADT groups. Sexual functioning in patients with MDD on antidepressants was also modestly improved after adding aripiprazole.

**Trial registration:**

ClinicalTrials.gov: NCT00095745 (November 9, 2004).

## Background

Aripiprazole is an atypical antipsychotic with partial agonist activity at dopamine D_2_, D_3_, and serotonin 5-HT_1A_ receptors, and antagonist activity at 5-HT_2A_ receptors [1-5]. Originally approved by the US Food and Drug Administration for treatment of schizophrenia and mania, aripiprazole was subsequently the first antipsychotic medication approved for use as an adjunctive treatment to antidepressants in adults with major depressive disorder (MDD) who have had an inadequate response to antidepressant treatment (ADT); the efficacy of adjunctive aripiprazole has been demonstrated in three double-blind, placebo-controlled trials [6-8]. Furthermore, a 52-week, open-label trial has also been conducted to assess the longer-term safety and tolerability of adjunctive aripiprazole in patients with an inadequate response to ADT [9]. In this study, aripiprazole augmentation was well tolerated, with an acceptable long-term safety and tolerability profile.

In the previously conducted, short-term, placebo-controlled trials [6-8], aripiprazole was given as adjunctive treatment to standard ADT with either selective serotonin reuptake inhibitors (SSRIs) or serotonin–norepinephrine reuptake inhibitors (SNRIs). Although the indication of adjunctive use of aripiprazole extends to antidepressants in general, controlled trials conducted to date have excluded patients receiving bupropion. This is important because, while the exact mechanism of action of bupropion remains unclear, it has been proposed that bupropion may act in part as a weak dopamine reuptake inhibitor [10,11] and, as such, the clinical effects of combining agents with pre- and postsynaptic effects on dopaminergic neurotransmission are unknown.

Beyond issues pertaining to safety, there are other reasons to examine the combination of bupropion and adjunctive aripiprazole. For example, whereas SSRIs and SNRIs can have adverse effects on sexual function [12], bupropion has been associated with neutral to positive effects on overall sexual function [13]. Bupropion has been shown to be as effective as the SSRIs for the treatment of MDD [14,15] with a lower risk of sexual dysfunction [15,16]. Among the newer generation antidepressants, bupropion also has a relatively low risk of weight gain [11,17].

Hypodopaminergic activity may be associated with diminished drive, amotivation, reduced cognitive function and lack of positive mood [18]. Moreover, a recent analysis suggests that patients with symptoms related to loss of interest or diminished activity experience diminished response to ADT treatments [19]. As such, patients with these symptoms may be disproportionately represented among patients with an inadequate response to ADTs and in need of additional treatment. Furthermore, given that aripiprazole is thought to have the lowest potential for metabolic disturbance and weight gain of the adjunctive atypicals commonly used in major depression [20], aripiprazole and bupropion may be a particularly appropriate treatment option for patients who are overweight or obese. Therefore, clinicians may choose to augment bupropion with aripiprazole to address specific symptoms thought to be associated with hypodopaminergic activity or to minimize particular adverse effects.

Aripiprazole as adjunctive treatment to bupropion was permitted in patients directly enrolling in the 52-week, open-label trial of aripiprazole augmentation [9]; data from these patients provided the opportunity to assess the safety and effectiveness of aripiprazole adjunctive to bupropion in patients with an inadequate response to antidepressant monotherapy. Furthermore, as this study included assessment of sexual functioning, the effect of aripiprazole adjunctive to bupropion on sexual function could also be evaluated. Thus, this post hoc analysis used data from this previously completed, 52-week, open-label study to assess the long-term safety, tolerability, and effectiveness of aripiprazole adjunctive to either bupropion or SSRIs/SNRIs in patients with MDD.

## Methods

### Study design and patients

This was a post hoc analysis of data from a subgroup of patients directly enrolled (did not participate in either of two previous double-blind, placebo-controlled trials) in a multicenter, 52-week, open-label study (ClinicalTrials.gov: NCT00095745) [9] of adjunctive aripiprazole to either bupropion or SSRIs/SNRIs. Data were pooled post hoc into the 2 ADT subgroups: bupropion plus aripiprazole, and all other SSRI/SNRI ADTs plus aripiprazole. Patients in this *de novo* subgroup were aged ≥18 years with *Diagnostic and Statistical Manual of Mental Disorders, Fourth Edition, Text Revision* criteria for a major depressive episode and a duration of current depressive episode of at least 8 weeks. All patients entered a 7- to 28-day pre-treatment screening phase consisting of a screening and baseline visit to assess study eligibility criteria and allow washout of prohibited concomitant pharmacotherapy. Full details of inclusion criteria have been presented previously [9]. Patients with a significant history of seizure disorder or other neurological disorder were excluded from *de novo* enrollment. Patients or their legal representatives provided written informed consent before participation. The study protocol was approved by the institutional review board at each site either through a local IRB or one of the following central IRBs: the Institutional Review Board, Inc., Laguna Hills, California; the Schulman Associates IRB, Inc., Cincinnati, Ohio; the IRB of the Office of Scientific Affairs, Philadelphia, Pennsylvania; and the Western Institutional Review Board, Olympia, Washington. The study was conducted in accordance with the ethical principles set forth in the Declaration of Helsinki.

Patients were eligible to enter 52 weeks of open-label treatment if they had an inadequate response (<50% improvement as assessed by the Massachusetts General Hospital Antidepressant Treatment Response Questionnaire) to at least one but no more than four ADT trials (each of at least 6 weeks duration at an adequate dose) [21]; a Montgomery-Åsberg Depression Rating Scale (MADRS) Total score >10 at baseline; and, in the opinion of the investigator, the presence of residual symptoms that might benefit from pharmacologic modification. Patients were also required to be currently taking one of the following ADTs at an adequate dose for a minimum of 6 weeks by the end of the screening phase: an SSRI (escitalopram, fluoxetine, sertraline, paroxetine or paroxetine controlled release [CR]); an SNRI (venlafaxine extended release [XR] or duloxetine); a norepinephrine–dopamine reuptake inhibitor (bupropion extended release [XL] or bupropion sustained release [SR]); or a tetracyclic antidepressant (mirtazapine).

All patients received ADT in accordance with current product labelling, with dose adjustments permitted within the recommended dose range. Adjunctive aripiprazole was initiated at 5 mg/day, and dosed in the range of 2–30 mg/day for patients receiving venlafaxine XR, escitalopram, mirtazapine or sertraline and 2–15 mg/day for patients on fluoxetine, paroxetine, duloxetine or bupropion (all CYP2D6 inhibitors).

### Study assessments

Safety was evaluated by monitoring adverse events (AEs) and vital signs (at baseline and each study visit), body weight (baseline, Weeks 26 and 52) and 12-lead electrocardiogram (ECG) (baseline, Weeks 8, 26 and 52). Laboratory tests, including fasting metabolic parameters, were conducted at open-label treatment baseline and Weeks 8, 26, 38 and 52. Effectiveness was assessed throughout the open-label treatment period using the Clinical Global Impressions–Severity (CGI-S) of illness (decrease in score signifies improvement in severity of illness) [22]. In addition, sexual function was assessed using the Massachusetts General Hospital Sexual Functioning Inventory (MGH-SFI) [23], a five-item scale that rates items (“Interest in Sex”, “Sexual Arousal”, “Ability to Achieve Orgasm”, “Ability to Maintain Erection” [males only] and “Sexual Satisfaction”) using a 6-point scale from 1 (good function, greater than normal) to 6 (poor function, totally absent). MGH-SFI items were rated at baseline and Weeks 8, 26 and 52. Overall improvement in sexual function since their last medication change was also assessed using a 6-point scale from 1 (very much improved) to 6 (much worse) at Weeks 8, 26 and 52.

### Analyses

For this post hoc analysis, data were pooled into the two ADT subgroups of bupropion plus aripiprazole and SSRI/SNRI plus aripiprazole. Safety analyses included all patients who received at least one dose of aripiprazole as adjunctive therapy to either bupropion or an SSRI/SNRI ADT, and analyses of effectiveness included all patients in the safety sample who had at least one CGI-S assessment during open-label treatment (efficacy sample). Summary statistics for change in effectiveness and safety rating scale scores are presented for patients treated with bupropion and for all other SSRI/SNRI ADTs combined. Data for patients receiving bupropion XL or bupropion SR were pooled into one group. Data for aripiprazole plus bupropion were compared only to the subset of patients receiving aripiprazole plus an SSRI/SNRI because all patients in the registered trials received aripiprazole as an adjunct to an SSRI/SNRI. This exploratory, post hoc subgroup analysis is limited by reduced power, increased variance, and an increased influence of chance [24]; thus, no formal statistical testing was planned. Analyses were based on the last observation carried forward (LOCF) or observed case (OC) data sets. The LOCF data set included data recorded at a given visit in the treatment phase or, if no observation was recorded at that visit, data carried forward from the previous visit in the treatment phase. The OC data set consisted of the actual observations at a given visit.

## Results

### Patient population and treatment

**Figure 1:**
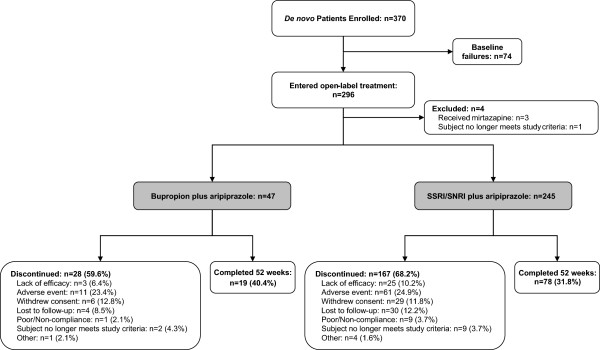
**Patient disposition (randomized sample).** SNRI, serotonin–norepinephrine reuptake inhibitor; SSRI, selective serotonin reuptake inhibitor.

Disposition, demographics, efficacy, safety, and tolerability in the overall study population were previously reported [9]. This study enrolled 370 *de novo* patients (i.e., they had not participated in one of two placebo-controlled trials) of whom 296 began the open-label treatment phase; the remaining 74 patients provided informed consent but did not meet eligibility criteria and were excluded from the open-label treatment protocol. Patient disposition of those treated with bupropion plus aripiprazole or an SSRI/SNRI plus aripiprazole is shown in Figure 1. Overall, 19 patients (40.4%) treated with bupropion plus aripiprazole and 78 patients (31.8%) treated with an SSRI/SNRI plus aripiprazole completed the 52-week treatment phase (p = 0.25). The most common reason for discontinuation of treatment in both treatment groups was AEs (23.4% for bupropion plus aripiprazole and 24.9% for an SSRI/SNRI plus aripiprazole; the proportions of patients who withdrew because of intolerable AEs did not differ significantly).

At entry into open-label treatment, the distribution of ADTs was as follows: bupropion XL/SR, n = 46 (15.8%); duloxetine, n = 7 (2.4%); escitalopram, n = 66 (22.7%); fluoxetine, n = 42 (14.4%); paroxetine, n = 28 (9.6%); paroxetine CR, n = 10 (3.4%); sertraline, n = 39 (13.4%); and venlafaxine XR, n = 50 (17.2%). The mean doses of ADT at study endpoint (the last day of each ADT dosing) were as follows: bupropion XL/SR 340 mg/day (n = 46); duloxetine 60 mg/day (n = 7); escitalopram 16.8 mg/day (n = 65); fluoxetine 41.2 mg/day (n = 42); paroxetine 34.4 mg/day (n = 27); paroxetine CR 29.3 mg/day (n = 10); sertraline 125 mg/day (n = 39); and venlafaxine XR 167 mg/day (n = 50). The mean dose of aripiprazole at study endpoint was 9.6 mg/day in patients receiving bupropion plus aripiprazole and 9.3 mg/day in patients receiving an SSRI/SNRI plus aripiprazole (n = 241; dosing information was missing for one patient).

Forty-six patients treated with bupropion plus aripiprazole and 242 treated with an SSRI/SNRI plus aripiprazole received at least one dose of study medication and were included in the safety sample. The baseline demographic characteristics of patients are presented in Table 1 and showed that demographic characteristics were similar between patients treated with bupropion plus aripiprazole and those treated with an SSRI/SNRI plus aripiprazole. The median time to discontinuation for all patients in the safety sample was 184.0 days. All 46 patients in the bupropion plus aripiprazole group had at least one CGI-S assessment during open-label treatment and were included in the efficacy sample, while the efficacy sample for an SSRI/SNRI plus aripiprazole included 236 patients (p = 0.25).

**Table 1:** Baseline demographics and disease severity by ADT group (safety sample)

**Baseline characteristic**	**Bupropion plus aripiprazole (n = 46)**	**SSRI/SNRI plus aripiprazole (n = 242)**
Age (years), mean (SD)	45.0 (12.9)	46.6 (12.6)
Gender, n (%)		
Females	30 (65.2)	166 (68.6)
Race, n (%)		
White	40 (87.0)	223 (92.1)
Black	4 (8.7)	12 (5.0)
Asian	0	2 (0.8)
Other	2 (4.3)	5 (2.1)
Weight (kg), mean (SD)	93.4 (23.2)	87.7 (22.4)
BMI (kg/m^2^), mean (SD)	32.5 (8.6)	31.2 (8.1)
CGI-S score, mean (SD)	4.2 (0.6)	4.2 (0.6)
MADRS Total score, mean (SD)	24.5 (4.8)	25.1 (6.0)
IDS-Self Rated Total score, mean (SD)	34.6 (9.4)	38.2 (10.5)
QIDS-Self Rated Total score, mean (SD)	13.5 (3.9)	14.6 (4.2)

CGI-S, Clinical Global Impressions–Severity score; IDS, Inventory of Depressive Symptomatology; MADRS, Montgomery–Åsberg Depression Rating Scale; QIDS, Quick Inventory of Depressive Symptomatology; SD, standard deviation; SNRI, serotonin–norepinephrine reuptake inhibitor; SSRI, selective serotonin reuptake inhibitor.

### Adverse events

During open-label treatment, 272 (94.4%) patients experienced at least one AE, and the rate of AEs was similar between bupropion plus aripiprazole (n = 45/46; 97.8%) and an SSRI/SNRI plus aripiprazole (n = 227/242; 93.8%). Treatment-emergent AEs, by ADT, that occurred at an incidence ≥10% in either ADT group are shown in Table 2. The most common AEs (>20% of patients in either group) were fatigue (26.1%) and somnolence (21.7%) with bupropion plus aripiprazole; 17.4% reported akathisia. Fatigue (23.6%) and akathisia (23.6%) were the most common AEs with an SSRI/SNRI plus aripiprazole, followed by weight increased (19.0%). The percentage of patients reporting weight increase as an AE was 8.7% and 19.0% with bupropion plus aripiprazole and an SSRI/SNRI plus aripiprazole, respectively. The incidence of erectile dysfunction and middle insomnia were both higher in patients treated with bupropion plus aripiprazole compared with those receiving an SSRI/SNRI plus aripiprazole. The overall pattern of AEs was generally comparable between SSRIs/SNRIs (Additional file 1).

**Table 2:** **Treatment-emergent AEs occurring in ≥10**% **of patients in either ADT group (safety sample)**

**AE, %**	**Bupropion plus aripiprazole (n = 46)**	**SSRI/SNRI plus aripiprazole (n = 242)**
Fatigue	26.1	23.6
Somnolence	21.7	18.2
Akathisia	17.4	23.6
Headache	17.4	15.3
Increased appetite	15.2	11.2
Insomnia	15.2	15.7
Arthralgia	13.0	3.3
Irritability	13.0	3.7
Middle insomnia	13.0	2.5
Upper respiratory tract infection	13.0	10.7
Erectile dysfunction	12.5	2.6
Anxiety	10.9	11.2
Asthenia	10.9	3.7
Back pain	10.9	5.4
Dry mouth	10.9	10.3
Nausea	10.9	11.6
Restlessness	10.9	12.4
Salivary hypersecretion	10.9	3.7
Tremor	10.9	8.7
Dizziness	8.7	10.3
Weight increased	8.7	19.0

ADT, antidepressant treatment; AE, adverse event; SNRI, serotonin–norepinephrine reuptake inhibitor; SSRI, selective serotonin reuptake inhibitor.

With respect to less common AEs, no patient treated with bupropion plus aripiprazole experienced seizures. Two SSRI/SNRI plus aripiprazole-treated patients reported seizures; a 53-year-old man with a remote history of a grand mal seizure reported a convulsion while receiving paroxetine 60 mg/day and aripiprazole 5 mg/day, and a 22-year-old female patient receiving escitalopram who had discontinued the study on Day 118 reported a convulsion on Day 155. Neither event was considered related to study medication.

### Weight change and metabolic abnormalities

Both ADT groups experienced a mean increase in weight over the course of treatment. Mean weight change at Week 52 (OC) was +3.1 kg for bupropion plus aripiprazole, and +2.4 kg for an SSRI/SNRI plus aripiprazole. Clinically significant weight gain (increase of ≥7% from baseline) occurred in 20.6% of bupropion plus aripiprazole and 26.2% of SSRI/SNRI plus aripiprazole patients at Week 52 (LOCF). Clinically significant weight loss (decrease of ≥7% from baseline) occurred in 5.9% and 7.1% of bupropion plus aripiprazole and SSRI/SNRI plus aripiprazole patients, respectively.

The metabolic findings for patients treated with bupropion plus aripiprazole and an SSRI/SNRI plus aripiprazole are shown in Table 3. The incidence of treatment-emergent, potentially clinically relevant fasting metabolic abnormalities and mean baseline and mean changes from baseline in fasting metabolic parameters between patients receiving bupropion plus aripiprazole and those receiving an SSRI/SNRI plus aripiprazole were variable. The incidence of treatment-emergent, potentially clinically relevant abnormalities in fasting glucose levels was lower (8.3% vs 17.4%) and the mean (SE) change in fasting glucose levels from baseline to Week 52 was also lower in patients treated with bupropion plus aripiprazole (1.4 [1.9] mg/dL) than with an SSRI/SNRI plus aripiprazole (2.7 [1.5] mg/dL). The incidence of treatment-emergent potentially clinically relevant abnormalities in fasting total cholesterol was also lower in patients treated with bupropion plus aripiprazole than with an SSRI/SNRI plus aripiprazole (25.0% vs 34.7%); however, there was no apparent difference in the mean change in fasting cholesterol levels from baseline to Week 52 between the two treatment groups.

**Table 3:** Fasting metabolic abnormalities by ADT group (safety sample)

**Metabolic measurement**	**Incidence of potentially clinically relevant fasting metabolic abnormalities, n (%)**		**Mean (SE) fasting metabolic parameters, mg/dL**			
	**Bupropion plus aripiprazole**	**SSRI/SNRI plus aripiprazole**	**Bupropion plus aripiprazole**^**1**^		**SSRI/SNRI plus aripiprazole**^**2**^	
			**Baseline**	**Change from baseline at Week 52**	**Baseline**	**Change from baseline at Week 52**
Glucose	3/36 (8.3)	35/201 (17.4)	95.0 (2.4)	1.4 (1.9)	94.4 (1.3)	2.7 (1.5)
Total cholesterol	9/36 (25.0)	70/202 (34.7)	209.6 (6.3)	-0.9 (6.6)	214.2 (3.2)	-0.9 (2.6)
LDL cholesterol	3/35 (8.6)	48/202 (23.8)	121.6 (5.3)	0.5 (5.7)	125.1 (2.9)	-3.1 (2.5)
HDL cholesterol	1/36 (2.8)	6/202 (3.0)	55.0 (3.0)	0.3 (1.4)	56.4 (1.2)	-0.5 (0.7)
Triglycerides						
Male	9/13 (69.2)	34/65 (52.3)	183.0 (36.6)	7.5 (13.7)	180.0 (13.9)	28.2 (19.9)
Female	15/23 (65.2)	100/137 (73.0)	157.5 (16.0)	-25.3 (8.9)	156.9 (9.7)	8.5 (7.1)

ADT, antidepressant treatment; HDL, high-density lipoprotein; LDL, low-density lipoprotein; SNRI, serotonin–norepinephrine reuptake inhibitor; SSRI, selective serotonin reuptake inhibitor.

Number of patients with both baseline and endpoint laboratory assessments: ^1^n = 32 (male, n = 11; female, n = 21); ^2^n = 181 (male, n = 58; female, n = 123) for all parameters except fasting glucose, where n = 179.

### Sexual function

**Figure 2:**
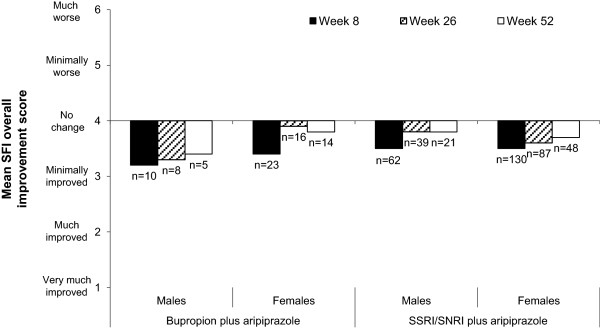
**Mean MGH-SFI overall improvement score over the course of treatment by ADT group and week (safety sample, OC).** ADT, antidepressant treatment; MGH-SFI, Massachusetts General Hospital Sexual Functioning Inventory; OC, observed case; SNRI, serotonin–norepinephrine reuptake inhibitor; SSRI, selective serotonin reuptake inhibitor.

Mean MGH-SFI overall improvement scores over the course of treatment for patients treated with bupropion plus aripiprazole and an SSRI/SNRI plus aripiprazole, by gender, are shown in Figure 2. In patients treated with either bupropion plus aripiprazole or an SSRI/SNRI plus aripiprazole, MGH-SFI rating of overall improvement indicated mild-to-moderate sexual dysfunction over the course of treatment, and at Week 52 (OC), the mean (SE) overall improvement score was 3.4 (0.5) in males and 3.8 (0.2) in females treated with bupropion plus aripiprazole and 3.8 (0.2) and 3.7 (0.1) in males and females treated with an SSRI/SNRI plus aripiprazole, respectively.

Mean change in MGH-SFI item scores from baseline to Week 52, by gender, for patients treated with bupropion plus aripiprazole and an SSRI/SNRI plus aripiprazole are shown in Table 4. Both male and female patients in both ADT groups generally experienced improvements in most items in both the OC and LOCF analyses.

**Table 4:** MGH-SFI item scores by ADT group and gender (safety sample)

**MGH-SFI item**	**Bupropion plus aripiprazole, mean (SE)**			**SSRI/SNRI plus aripiprazole, mean (SE)**		
	**Baseline**	**Change from baseline at Week 52 (OC)**	**Change from baseline at Week 52 (LOCF)**	**Baseline**	**Change from baseline at Week 52 (OC)**	**Change from baseline at Week 52 (LOCF)**
Males, n	13	5	13	69	21	69
Interest in sex	2.8 (0.3)	-0.2 (0.9)	0.0 (0.4)	3.8 (0.2)	-0.5 (0.4)	-0.3 (0.2)
Ability to get sexually aroused	3.1 (0.3)	0.0 (0.8)	-0.1 (0.3)	3.9 (0.2)	-0.8 (0.3)	-0.3 (0.2)
Ability to achieve orgasm	3.2 (0.4)	-0.4 (1.1)	-0.2 (0.4)	4.0 (0.2)	-0.7 (0.4)	-0.4 (0.2)
Ability to get erection	3.7 (0.4)	-1.2 (0.4)	-0.6 (0.2)	3.7 (0.2)	-0.3 (0.4)	-0.2 (0.2)
Overall sexual satisfaction	3.9 (0.4)	-1.4 (0.2)	-0.7 (0.3)	4.1 (0.2)	-0.6 (0.3)	-0.4 (0.2)
Females, n	24	14	24	147	48	147
Interest in sex	3.9 (0.3)	-0.5 (0.3)	-0.5 (0.3)	4.6 (0.1)	-0.9 (0.2)	-0.9 (0.1)
Ability to get sexually aroused	4.0 (0.3)	-0.4 (0.5)	-0.5 (0.3)	4.6 (0.1)	-0.9 (0.2)	-0.7 (0.1)
Ability to achieve orgasm	3.9 (0.4)	-0.3 (0.5)	-0.3 (0.3)	4.6 (0.1)	-0.8 (0.2)	-0.7 (0.1)
Overall sexual satisfaction	4.1 (0.3)	-0.8 (0.4)	-0.7 (0.3)	4.7 (0.1)	-1.1 (0.2)	-0.8 (0.1)

ADT, antidepressant treatment; LOCF, last observation carried forward; MGH-SFI, Massachusetts General Hospital Sexual Functioning Inventory; OC, observed case; SNRI, serotonin–norepinephrine reuptake inhibitor; SSRI, selective serotonin reuptake inhibitor.

MGH-SFI item scores range from 1 (greater than normal) to 6 (totally absent). A negative change score signifies improvement.

### Vital signs and laboratory findings

The incidence of potentially clinically relevant electrocardiographic abnormalities was low with both bupropion plus aripiprazole and an SSRI/SNRI plus aripiprazole. The most common ECG abnormality was QT_C_ (Bazett) prolongation in patients treated with an SSRI/SNRI plus aripiprazole (8.8%), with 1.8% of patients experiencing the abnormality at more than one time point; all other abnormalities occurred in <3% of patients. Only one ECG abnormality was reported in bupropion plus aripiprazole-treated patients: one case of QT_C_ (Bazett) prolongation (2.4%). The incidence of potentially clinically relevant serum chemistry, hematology and electrolyte measurements was also low in both ADT groups. Exceptions to this were the incidence of potentially clinically relevant prolactin abnormalities (14.6% for bupropion plus aripiprazole and 16.0% for an SSRI/SNRI plus aripiprazole), hemoglobin A1C (7.3% for bupropion plus aripiprazole and 10.2% for an SSRI/SNRI plus aripiprazole) and uric acid levels in females (11.1% for bupropion plus aripiprazole and 2.0% for an SSRI/SNRI plus aripiprazole).

### Symptom severity

**Figure 3:**
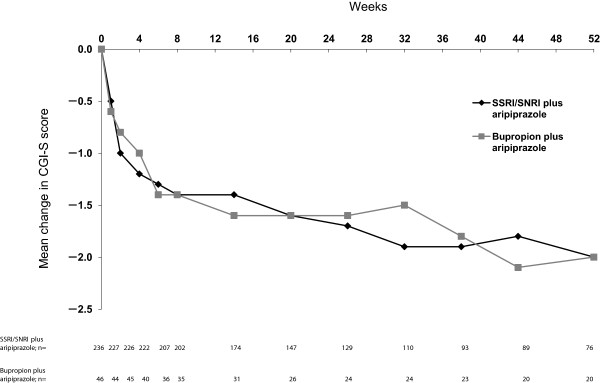
**Mean change in CGI-S from baseline to Week 52 by ADT group and week (efficacy sample, OC).** Mean (SE) baseline CGI-S scores: bupropion plus aripiprazole, 4.2 (0.1); SSRI/SNRI plus aripiprazole, 4.2 (0.0). ADT, antidepressant treatment; CGI-S, Clinical Global Impressions–Severity score; OC, observed case; SNRI, serotonin–norepinephrine reuptake inhibitor; SSRI, selective serotonin reuptake inhibitor.

Improvement in symptom severity, as measured by mean change in CGI-S from baseline to Week 52 for bupropion plus aripiprazole and an SSRI/SNRI plus aripiprazole patients are shown in Figure 3 (OC analysis). For both ADT groups, continued improvement in CGI-S scores was seen over the course of treatment. At Week 52 (OC), the mean change in CGI-S scores was -2.0 points in bupropion plus aripiprazole patients (n = 20) and -2.0 points in SSRI/SNRI plus aripiprazole patients (n = 76). At Week 52 (LOCF), the mean change in CGI-S scores was -1.4 points in bupropion plus aripiprazole patients (n = 46) and -1.5 points in SSRI/SNRI plus aripiprazole patients (n = 236).

## Discussion

Aripiprazole is an atypical antipsychotic with a unique pharmacological profile, and while the exact mechanism of action of atypical antipsychotic augmentation is not well understood, it is possible that the pharmacological profile of aripiprazole may be particularly effective as an augmentation agent in depression. In this study, improvement in depressive symptoms occurred when aripiprazole was added to either bupropion or an SSRI/SNRI and symptom improvement was maintained over a 52-week treatment period at a similar level with both ADT groups. Thus, combining two agents with effects on dopaminergic activity does not appear to inhibit the benefits of aripiprazole as an augmentation agent. However, the pharmacodynamic effects of concomitant use of aripiprazole and bupropion on dopamine neurotransmission warrant further investigation.

With respect to safety, this is to our knowledge the largest prospective study of the use of aripiprazole adjunctive to bupropion conducted to date. Aripiprazole augmentation of bupropion was not associated with any unexpected AEs and had a long-term safety profile that was comparable to that of aripiprazole augmentation of SSRIs/SNRIs in patients with MDD who had experienced an inadequate response to ADT monotherapy. Importantly, there was no evidence of an adverse synergistic or additive effect on dopamine associated with co-administration of aripiprazole and bupropion, compared with other aripiprazole/ADT combinations, and reasons for discontinuation were similar between adjunctive ADT groups. Notably, rates of akathisia were not higher with bupropion plus aripiprazole than with aripiprazole adjunctive to SSRI/SNRI ADTs. Seizures are one of the neurologic side effects reported with bupropion [10,11]. While no seizures were reported with bupropion plus aripiprazole, doses of bupropion were low, as recommended to minimize the risk of seizures [11], and patients with a significant history of seizure disorder were excluded.

Both bupropion and SSRIs/SNRIs adjunctive with aripiprazole were associated with a mean increase in weight over the 52 weeks of treatment, with no apparent association between type of antidepressant and the extent of weight gain. Although it is difficult to establish the relative contribution of aripiprazole to the weight gain reported here given that long-term administration of antidepressant medications is also associated with medically relevant weight gain [17], all patients receiving adjunctive aripiprazole, regardless of the particular antidepressant, should be monitored for weight gain and proactively managed when it occurs.

Metabolic disturbances associated with medication are particularly important during long-term treatment. The incidence of treatment-emergent, potentially clinically relevant abnormalities in fasting glucose levels was lower in patients receiving bupropion plus aripiprazole than an SSRI/SNRI plus aripiprazole and mean changes in glucose levels tended to favor the group treated with bupropion plus aripiprazole. The incidence of treatment-emergent potentially clinically relevant abnormalities in fasting total cholesterol was also lower for patients receiving bupropion plus aripiprazole than an SSRI/SNRI plus aripiprazole, although there was no difference in mean change from baseline between the treatment groups. Findings for other metabolic parameters are harder to interpret. However, the relatively favorable metabolic profile of aripiprazole among atypical antipsychotics support the possibility that changes in metabolic parameters stem from SSRI/SNRI treatment in this patient group [20] or their interaction with aripiprazole.

Sexual dysfunction is common in patients with MDD and is an important concern during treatment, especially as some antidepressant therapies can contribute to the emergence or exacerbation of sexual dysfunction. Although variability exists within the therapeutic classes, SSRIs and SNRIs generally worsen sexual function [12], while norepinephrine–dopamine reuptake inhibitors, such as bupropion, are associated with neutral-to-positive effects on overall sexual function [13]. Indeed, bupropion is recommended as an ADT in patients that experience sexual side effects with an SSRI medication [11]. As such, it is not surprising that baseline levels of sexual function in this analysis were slightly worse in patients who received an SSRI/SNRI plus aripiprazole than patients receiving bupropion plus aripiprazole. In this study, sexual function was modestly improved after the addition of aripiprazole to either bupropion or an SSRI/SNRI, in both men and women, with a similar pattern/level of change across all ADT medications. This finding would be expected given that the pharmacologic profile of aripiprazole would not be expected to be associated with sexual dysfunction [1-5]. Aripiprazole adjunctive to ADT has previously been shown to have some beneficial effects on sexual function in patients with MDD who respond inadequately to standard ADT in short-term studies [25]. The findings reported here represent the first data exploring the long-term impact of adjunctive aripiprazole on sexual function and, importantly, show that aripiprazole/ADT combinations were not associated with a worsening of sexual function in this patient population. Although erectile dysfunction as an AE was more commonly reported with bupropion plus aripiprazole than an SSRI/SNRI plus aripiprazole, MGH-SFI ratings of “Ability to get erection” did not support this finding, and in fact, were contradictory. Finally, while the relationship between improvement in sexual functioning and improvement in depression symptoms was not investigated in this study, previous analysis of short-term data has shown that the beneficial effects of adjunctive aripiprazole on sexual functioning were independent of the improvement in depressive symptoms [25].

This analysis explored the effects of aripiprazole as adjunctive therapy to bupropion. One of the strengths of this analysis is that the large patient population included in the original multicenter study consisted of a relatively large subgroup of patients treated with bupropion plus aripiprazole, permitting further analysis of this data set; previously published research on the use of aripiprazole adjunctive to bupropion is limited to four case reports [26]. However, a number of limitations of this analysis should be considered when interpreting the findings. This was an open-label study with no control group, which leaves uncertain the extent to which the observed effects were due to the medication. As several of the more commonly observed, treatment-emergent AEs in this study could be viewed as depressive symptoms (ie, fatigue and somnolence), it is at least possible that these were symptoms, not AEs, and this cannot be resolved in the absence of a randomized control group. This was a post hoc analysis and pooling of patients into the ADT subgroups was retrospective. Thus, this study was not specifically designed or powered to detect difference between ADT subgroups and, as such, the findings should be considered preliminary. Assessment of effectiveness did not include a specific rating scale for depression and was based on observed cases. It should also be considered that due to treatment discontinuation, the sample size of the ADT groups declines throughout this 1-year trial. Finally, assignment to treatment with ADT was based on investigator judgement and, as with any clinical series that does not use random treatment assignment, has the potential to lead to inherent differences in patients by antidepressant group and subsequent bias. However, assignment to treatment based on antidepressant history and clinical judgment is more closely representative of real-world practice, thus strengthening the real-world validity of the findings reported.

## Conclusions

The addition of aripiprazole augmentation to bupropion was not associated with any unexpected AEs and the overall tolerability of adjunctive aripiprazole was similar between bupropion and SSRI/SNRI. The addition of aripiprazole augmentation to antidepressant therapy results in improvements in depression symptoms and sexual function in patients with MDD who had not responded to treatment with one or more ADTs and the benefits did not vary when added to the norepinephrine–dopamine reuptake inhibitor bupropion or an SSRI/SNRI, the latter of which is now a well-established combination.

## Abbreviations

ADT: Antidepressant treatment; AE: Adverse event; CGI-S: Clinical Global Impressions–Severity score; CR: Controlled release; ECG: Electrocardiogram; LOCF: Last observation carried forward; MADRS: Montgomery-Åsberg Depression Rating Scale; MDD: Major depressive disorder; MGH-SFI: Massachusetts General Hospital Sexual Functioning Inventory; OC: Observed case; SNRI: Serotonin–norepinephrine reuptake inhibitor; SR: Sustained release; SSRI: Selective serotonin reuptake inhibitor; XL: Extended release (bupropion); XR: Extended release (venlafaxine).

## Competing interests

During the past 5 years, Anita H. Clayton has received research grants from BioSante Pharmaceuticals, Inc., Boehringer-Ingelheim, Bristol-Myers Squibb, Eli Lilly and Company, Forest Research Institute, Inc., Novartis, Palatin Technologies, Pfizer, Inc., Repligen Corporation, sanofi-aventis, Takeda, and Trimel Pharmaceuticals; has been an advisor/consultant to Apricus Biosciences, Inc., Astra Zeneca, Boehringer-Ingelheim, Bristol-Myers Squibb, Concert Pharmaceuticals, Eli Lilly and Company, Euthymics, Forest Research Institute, Inc., Labopharm, Inc, Lundbeck, New England Research Institutes, Inc., Novartis Pharmaceuticals, Palatin Technologies, Pfizer, Inc., PGxHealth, sanofi-aventis, S1 Biopharmaceuticals, Inc., Sprout Pharmaceuticals, Takeda Global Research & Development, TransTech Pharma, Inc., Trimel Pharmaceuticals, and Wyeth; has participated in a disease-state speakers bureau for Boehringer-Ingelheim and Eli Lilly and Company; has received royalties/copyright fees from Ballantine Books/Random House, Changes in Sexual Functioning Questionnaire, Guilford Publications, and Healthcare Technology Systems, Inc.; has held shares/restricted stock units in Euthymics and S1 Biopharmaceuticals, Inc.; and has had a US provisional patent application (Compositions and Methods for Diagnosing and Monitoring the Development of Tardive Dyskinesia, Inventors: Atmaram Yarlagadda and Anita Clayton, Patent Foundation University of Virginia).

During the past 3 years, Michael E. Thase has been an advisor/consultant for Aldolar, Alkermes, AstraZeneca, Bristol-Myers Squibb., Eli Lilly & Co., Forest Pharmaceuticals (including PGx Health), Johnson & Johnson (Janssen Pharmaceutica), Lundbeck, MedAvante, Inc., Merck, Neuronetics, Inc., Otsuka, Pfizer Inc., PharmaNeuroBoost, Rexahn, Roche, Shire US, Inc., and Takeda. Dr Thase has received honoraria for talks from AstraZeneca, Bristol-Myers Squibb, Eli Lilly & Co., Merck, and Pfizer. Dr Thase has received research grants from the Agency for Health Care Research and Quality, Alkermes, the National Institute of Mental Health, Eli Lilly & Co., Forest Pharmaceuticals, GlaxoSmithKline, Otsuka, PharmaNeuroBoost, and Roche. He has equity holdings in MedAvante, Inc. and receives income from royalties from American Psychiatric Publishing, Inc., Guilford Publications, and Herald House. Dr Thase’s wife is an employee of Embryon, Inc.

Robert M. Berman, Zachary J. Cain, Ronald Marcus, and John Sheehan are employees of Bristol-Myers Squibb; Sabrina Vogel Marler was an employee of Bristol-Myers Squibb when these post hoc analyses were conducted. John Sheehan is a former employee of Janssen Pharmaceuticals, Inc.

Ross A. Baker is an employee of Otsuka Pharmaceutical Development & Commercialization; Robert A. Forbes was an employee of Otsuka Pharmaceutical Development & Commercialization when these post hoc analyses were conducted.

## Authors’ contributions

AHC, RAB, JJS, SVM and MET contributed to the conception and design of the analysis. MET was a study investigator and participated in data collection. RAB and JJS managed the analyses. SVM undertook the statistical analysis. All authors contributed to the analysis and interpretation of data, critically reviewed all drafts to provide important intellectual content, and made the decision to submit the manuscript. All authors have approved the final manuscript.

## Authors’ information

John J. Sheehan, Sabrina Vogel Marler, Ronald Marcus, and Robert M. Berman were employees of Bristol-Myers Squibb when this study was performed.

## Supplementary Material

Additional file 1 Treatment-emergent AEs occurring in ≥10% of patients in any ADT group (safety sample). Click here for file
