# Supplementary material for: Comparison of adjunctive use of aripiprazole with bupropion or selective serotonin reuptake inhibitors/serotonin–norepinephrine reuptake inhibitors: analysis of patients beginning adjunctive treatment in a 52-week, open-label study
Source: BMC Res Notes. 2014 Jul 18;7:459. doi: 10.1186/1756-0500-7-459 (PMC4119176; doi:10.1186/1756-0500-7-459)
Supplement: Additional file 1 — Treatment-emergent AEs occurring in ≥10% of patients in any ADT group (safety sample). [file 1756-0500-7-459-S1.docx]

Treatment-emergent AEs occurring in ≥10% of patients in any ADT group (safety sample).

| **AE, %** | **Bupropion + aripiprazole (n=46)** | **SSRI/SNRI + aripiprazole**  **(n=242)** | **Venlafaxine XR + aripiprazole (n=50)** | **Escitalopram + aripiprazole (n=66)** | **Fluoxetine + aripiprazole**  **(n=42)** | **Sertraline + aripiprazole (n=39)** | **Paroxetine + aripiprazole (n=38)** | **Duloxetine + aripiprazole (n=7)** |
| --- | --- | --- | --- | --- | --- | --- | --- | --- |
| Akathisia | 17.4 | 23.6 | 24.0 | 28.8 | 23.8 | 17.9 | 21.1 | 14.3 |
| Fatigue | 26.1 | 23.6 | 34.0 | 22.7 | 14.3 | 25.6 | 21.1 | 14.3 |
| Somnolence | 21.7 | 18.2 | 16.0 | 24.2 | 21.4 | 20.5 | 5.3 | 14.3 |
| Weight increased | 8.7 | 19.0 | 18.0 | 16.7 | 31.0 | 12.8 | 15.8 | 28.6 |
| Insomnia | 15.2 | 15.7 | 14.0 | 18.2 | 14.3 | 23.1 | 7.9 | 14.3 |
| Headache | 17.4 | 15.3 | 14.0 | 10.6 | 11.9 | 20.5 | 21.1 | 28.6 |
| Restlessness | 10.9 | 12.4 | 10.0 | 15.2 | 11.9 | 15.4 | 7.9 | 14.3 |
| Nausea | 10.9 | 11.6 | 6.0 | 12.1 | 16.7 | 10.3 | 13.2 | 14.3 |
| Increased appetite | 15.2 | 11.2 | 10.0 | 9.1 | 14.3 | 12.8 | 10.5 | 14.3 |
| Anxiety | 10.9 | 11.2 | 8.0 | 10.6 | 9.5 | 17.9 | 10.5 | 14.3 |
| Back pain | 10.9 | 5.4 | 2.0 | 10.6 | 2.4 | 5.1 | 5.3 | 0.0 |
| Tremor | 10.9 | 8.7 | 10.0 | 10.6 | 11.9 | 5.1 | 5.3 | 0.0 |
| Erectile dysfunction | 12.5 | 2.6 | 0.0 | 10.5 | 0.0 | 0.0 | 0.0 | 0.0 |
| Upper respiratory tract infection | 13.0 | 10.7 | 12.0 | 7.6 | 9.5 | 20.5 | 7.9 | 0.0 |
| Sedation | 8.7 | 9.1 | 6.0 | 7.6 | 9.5 | 12.8 | 13.2 | 0.0 |
| Dizziness | 8.7 | 10.3 | 4.0 | 9.1 | 16.7 | 12.8 | 10.5 | 14.3 |
| Diarrhea | 4.3 | 8.7 | 8.0 | 6.1 | 9.5 | 15.4 | 7.9 | 0.0 |
| Dry mouth | 10.9 | 10.3 | 12.0 | 6.1 | 19.0 | 12.8 | 2.6 | 14.3 |
| Constipation | 4.3 | 6.6 | 10.0 | 4.5 | 9.5 | 0.0 | 10.5 | 0.0 |
| Irritability | 13.0 | 3.7 | 6.0 | 4.5 | 0.0 | 7.7 | 0.0 | 0.0 |
| Asthenia | 10.9 | 3.7 | 8.0 | 4.5 | 2.4 | 0.0 | 2.6 | 0.0 |
| Nasopharyngitis | 8.7 | 7.0 | 14.0 | 3.0 | 7.1 | 10.3 | 2.6 | 0.0 |
| Arthralgia | 13.0 | 3.3 | 2.0 | 3.0 | 2.4 | 5.1 | 5.3 | 0.0 |
| Urinary tract infection | 4.3 | 7.0 | 14.0 | 1.5 | 14.3 | 7.7 | 0.0 | 0.0 |
| Salivary hypersecretion | 10.9 | 3.7 | 4.0 | 1.5 | 11.9 | 0.0 | 0.0 | 14.3 |
| Hyperhidrosis | 4.3 | 4.1 | 2.0 | 1.5 | 2.4 | 0.0 | 15.8 | 14.3 |
| Middle insomnia | 13.0 | 2.5 | 4.0 | 0.0 | 2.4 | 7.7 | 0.0 | 0.0 |

Shaded boxes represent AEs occurring at an incidence ≥10%.
ADT, antidepressant treatment; AE, adverse event; SNRI, serotonin–norepinephrine reuptake inhibitor; SSRI, selective serotonin reuptake inhibitor; XR, extended release.
